# PW02-016 - 41 cases of TRAPS, a rare autoinflammatory disease

**DOI:** 10.1186/1546-0096-11-S1-A156

**Published:** 2013-11-08

**Authors:** M-C Chastang, M Desjonqueres, V Hentgen, P Quartier Dit Maire, G Grateau, I Kone-Paut, B Bader-Meunier, C Wouters, I Lemelle, P Cochat

**Affiliations:** 1 Hopital Femme Mère Enfant CHU Lyon, Lyon, France; 2 CH Versaille, Le Chesnay, France; 3 Hopital Necker Enfants Malades, Paris; 4 CH Tenon, Paris; 5 Bicetre, Le Kremlin Bicetre, France; 6 University Hospital Leuven, Leuven, Belgium; 7 Brabois Enfant, Nancy, France

## Introduction

TRAPS (TNF receptor associated periodic syndrome) is a rare autoinflammatory disease that can affect children and adults. It is caused by the mutation of TNFRSF1A encoding for the TNF receptor. The main complication is amyloidosis.

## Objectives

The aim is to increase knowledge about the disease in order to make the diagnostic easier. Another purpose is to analyse the biotherapy treatment in TRAPS.

## Methods

It consists in a retrospective descriptive multicentre study in French and Belgian hospitals. Data has been directly collected thanks to patient files.

Inclusion criteria are: presence of TNFRAF1A mutation, recurrent symptoms.

## Results

We have included 25 children and 16 adults (isolated cases and 9 families), coming from France (45%), south of Europe (22%), north of Europe (10%), Maghreb (9%), east of Europe (6%).

19,5% of the patients have had an appendectomy. 26 patients have recurrent fever in their family, among which 22 have TRAPS. Two kids have homozygous mutation for MEFV and one heterozygous.

The disease starts mainly before the age of 5 years (61,1%) but for 13,5%, it begins in adulthood. The average time of diagnosis (delay between first symptoms and diagnosis) is 12,9 years.

51% of R92Q heterozygous mutation, 10% of T50M, 7% de L67P, 5% C29S, 5% C43S have been encountered. 2% of the patients have R92Q homozygous, 2% Q82R and R92Q heterozygous.

The seizures occur 9,7 times a year on average (<1 to 48 times a year), last 10,8 days on average (1 to 49 days). A trigger exists in 43.9% of the cases.

78% have rheumatologic symptoms, 70,7% arthralgia (mainly knees, spine, elbows), 22% arthritis (small and big joints). 24,4% have chest pain, 7,3%serositis. Dermatological symptoms (70,7%) are frequent (56,1% rash). Lots of patients have abdominal pain (70,7%), myalgia (65,7%), asthenia (48,8%). Headache is present in 39% of this population. Only 3 patients have periorbital oedema.

Between the seizures there is no symptomatology, but in 24% of the cases inflammatory syndrome persists. We note the interest to dose the Serum Amyloid A to detect the activity of disease between the attacks.

The screening of proteinuria was positive in 29% of the cases but no amyloidis has been reported.

No interesting correlation was found between genotype and phenotype.

Corticosteroids were used for treatment of seizures. Only 9 patients were treated by biotherapy. Etanercept was efficient in a first time, but not always in the long term. Anakinra always allowed remission.

## Conclusion

77%of this population of TRAPS has 3 symptoms among arthralgia, rash, abdominal pain, myalgia, asthenia and headache. Etanercept is not always efficient and Anakinra is probably a good alternative for the treatment. The inscription of the patients in autoinflammatory disease registers would allow a better knowledge of TRAPS.

## Disclosure of interest

None declared.

